# Chronic Non-Communicable Disease and Healthcare Access in Middle-Aged and Older Women Living in Soweto, South Africa

**DOI:** 10.1371/journal.pone.0078800

**Published:** 2013-10-29

**Authors:** Daniel Lopes Ibanez-Gonzalez, Shane A. Norris

**Affiliations:** Developmental Pathways for Health Research Unit, University of the Witwatersrand, Johannesburg, South Africa; Wits Reproductive Health and HIV Institute, South Africa

## Abstract

The aim of the current study was to describe the healthcare access, beliefs, and practices of middle-aged and older women residing in Soweto. This is a cross-sectional study of the primary (female) caregivers of the Birth to Twenty Cohort, based in Soweto, South Africa. The study instrument was administered to 1 102 caregivers as part of routine annual data collection. Over half the respondents (50.7%) reported having at least one chronic non-communicable disease (CND), only a small portion (33.3%) of whom reported accessing a healthcare service in the last 6 months. Reported availability of private medical practice and government clinics was high (75.1% and 61.5% respectively). The low utilisation of healthcare services by women with CND is a concern in terms of healthcare management. There is a need to further investigate how healthcare beliefs are formed, as well as the feasibility of programmes to support the ongoing management of CND in Soweto.

## Introduction

Since the dis-establishment of racially segregated public healthcare services in South Africa, substantial progress has been made in the formulation of healthcare policy and the integration of the healthcare system [1]. These efforts have been based on a commitment to a primary health care (PHC) approach in South Africa with an emphasis on full community participation in the planning, provision, control and monitoring of healthcare services within an integrated national health system (NHS) [2]. At the same time, the full achievement of a PHC oriented national health system has been hampered by a number of obstacles, including health worker shortages, inequities in resource distribution, shortcomings in health leadership and a protracted health transition [3]. The rise of chronic non-communicable disease (CND) in this context is projected to increase the demand for healthcare services, accounting for 28% of the total burden of disease measured by disability adjusted life years in 2004 [4].

In the context of a changing healthcare system and an increasing burden of CND, public health researchers and policy makers have increasingly recognised the need to focus on the “demand-side” of healthcare access, including the study of the preferences of healthcare users [5]. The need to focus on user preferences extends beyond the private-public domain, into the domain of healthcare beliefs regarding the efficacies of different healthcare modalities. The aim of our study was to describe the healthcare access, beliefs, and practices of middle-aged and old women residing in Soweto, South Africa.

## Methods

### Study population

This is a cross-sectional study of the female caregivers of the Birth to Twenty (Bt20) cohort. The Bt20 cohort started in 1989 with pilot studies to test the feasibility of a long-term follow-up study of children's health and wellbeing [6]. Women were enrolled in their second and third trimester of pregnancy through public health facilities. Singleton children (n = 3 273) born between April and June 1990 and resident for at least 6 months in the municipal area of Soweto-Johannesburg were enrolled into the birth cohort [7], [8]. The study is currently in contact with 70% of the cohort.

Between October 2008 and June 2010 the research team administered a semi-structured questionnaire to the primary caregivers of the Bt20 cohort in their home language (mainly Sotho and Zulu), after obtaining written consent. The study protocol was approved by the Human Research Ethics Committee (Medical) of the University of the Witwatersrand (M090235).

### Study protocol

The questionnaire included a number of domains. The demographic section included demographic measures; asset-based socio-economic status; employment status; and religious affiliation and adherence. The general healthcare access section included measures of availability and affordability of healthcare services; medical aid; and perceived obstacles to accessing public healthcare. The specific healthcare access section included measures of recent illness and healthcare services accessed, as well as experiences of the healthcare visit. The health-seeking behaviour section included measures of reliance on family and community networks for accessing healthcare and measures of patient strategy when interacting with formal healthcare systems. This section also included measures of use of traditional healers and self-rated assessments of the efficacy of traditional healers. The final section on CND included measures of disease prevalence and the use of lifelong medication. These measures, as well as some of the measures on general and specific healthcare access were adapted from the Adult Questionnaire of the South African Demographic and Health Survey [9].

The research team piloted the questionnaire in October of 2008, and administered the final questionnaire from November 2008 to June 2010. The questionnaire took less than an hour to complete. The interviews were conducted in the homes of the study participants in their preferred language by trained and experienced fieldworkers. The home setting coupled with the long-standing rapport and trust with experienced fieldworkers over the 18 years of the study facilitated more frank responses.

### Statistical analyses

The principal investigators descriptively analysed the data using STATA/IC 10.0. A series of Pearson's Chi-squared analyses described the basic relationships between demographic and disease-state characteristics and health-seeking behaviour.

## Results

### Study Demographics

A total of 1 102 women participated in the study. The demographic profile of the study sample describes a low resource population with a high prevalence of CND (Table 1).

**Table 1:** Population Characteristics.

Variable	Category	N	%
Age	30 to 39	266	24.1%
	40 to 49	589	53.5%
	50 to 65	245	22.2%
	Missing	2	0.2%
	**Total**	**1 102**	**100**%
Socio-economic indicators (Ownership)	Indoor hot and cold water (n = 1102)	405	36.8%
	Flush toilet inside (n = 1102)	538	48.8%
	Live in house (n = 1101)	980	88.9%
	Own house (n = 1101)	885	80.3%
	Own motor vehicle (n = 1099)	348	31.6%
	High SES (4–5 items)	190	17.2
	Medium SES (2–3 items)	496	45.0
	Low SES (0–1 items)	411	37.3
	Missing	5	0.5
	**Total**	**1 102**	**100**%
Employment	Formal or informal paid labour	582	52.8%
	Housewife/pensioner/unemployed	519	47.1%
	Missing	1	0.1%
	**Total**	**1 102**	**100**%
Self-reported chronic disease	No chronic disease	543	49.3%
	One chronic disease	339	30.8%
	More than one chronic disease	219	19.9%
	Missing	1	0.1%
	**Total**	**1 102**	**100**%

The large majority of participants lived in and owned houses, which, however, were without sole access to indoor hot and cold water or indoor flush toilets (Table 1). Over one third (37.3%) of the population could be categorised as having a low socio-economic status, defined as access to only one or less of 5 socio-economic items, including indoor running hot and cold water, indoor flush toilets, living in a house, owning a house, and owning a motor vehicle. Over half (52.8%) of the population were engaged in some form of paid employment, with a significant positive correlation between employment and socio-economic status (p<0.05).

Slightly over half the respondents (50.7%) reported having at least one CND. Of those that reported a CND 32.5% had high blood pressure, 12.8% had arthritis, 8.7% had high blood cholesterol, and 6.5% had diabetes. The remaining proportion of responses was distributed over a variety of CNDs, which individually constituted less than 5% of the total responses. Of those reporting CND, over two-thirds (69%) used prescribed lifelong medication on a regular basis. Respondents reported an average duration of CND of 8 and a half years, with an average self-reported delay of treatment of about 9 and a half months.

### Access to Healthcare Services

Access to healthcare services was determined by measures of availability and affordability. The availability of healthcare services was determined by whether a healthcare service provider was available within a 2 km radius or 20 minute walking distance from the respondent. Those who reported availability of healthcare services were asked whether they felt the services were affordable for them.

Private medical practices were more easily available than government clinics (75.1% and 61.5% respectively), although not as affordable (59.1% and 83.6% respectively) (Table 2). While sangomas were easily available for almost a third of the respondents (32.1%), they were reported as the least affordable (28.2%) of the formal and informal healthcare services.

**Table 2:** Access to Healthcare Services in Soweto.

	Proportion of respondents reporting availability within 2 km radius	Proportion of respondents reporting affordability (if available)
Private medical practice (n = 1 102)	75.1%	59.1%
Private hospital or clinic (n = 1 102)	7.4%	52.4%
Government or community clinic (n = 1 102)	61.5%	83.6%
Government hospital (n = 1 102)	3.9%	88.4%
Community organisation (n = 1 102)	30%	63.8%
Pharmacist (n = 1 101)	37%	72.9%
Sangoma or traditional healer (n = 1 101)	32.1%	28.2%
Herbalist (n = 1 101)	15%	63.9%
Faith Healer or priest (n = 1 102)	18.2%	57.5%

An additive index of formal healthcare services, including private doctors, hospitals and clinics and public hospitals and clinics, shows that around 85.8% of the respondents had at least one type of healthcare service available within a 2 km radius of their homes. Of this group 18% felt that these services were unaffordable. Around 17.4% of respondents had medical aid.

### Experiences of Healthcare Services

In the context of a high rate of CND in the study population, it is surprising that slightly less than a quarter of the respondents (24.3%) reported an illness within the last 6 months which obliged them to access healthcare services. The precise phrasing of the question was: ‘Have you had any illness or condition in the last 6 months so that you have had to seek treatment or healthcare?’ Those with CND were significantly more likely to report such illnesses (p<0.05), with around a third (33.3%) actually doing so.

All participants reporting an illness in the last 6 months (n = 268) were grouped into the following categories:

1. Trauma, including accidents, burns, and operations;
2. General ailments, including problems with ears, teeth, sinuses etc.,
3. Infectious diseases, such as bronchitis, TB, HIV, and infections; and
4. Chronic diseases, including osteoporosis, hypertension, diabetes, and arthritis.

Most of those respondents reporting an illness in the last 6 months had general ailments (35.8%), followed by chronic diseases (34.3%). Around 22% reported infectious diseases in the last 6 months, while 7.8% reported trauma.

Over half the respondents (60.5%) who reported a disease or condition treated their illnesses at a public healthcare facility (defined as a government clinic or hospital), with the remainder utilising some form of private healthcare service (private medical practice, private hospital or clinic, self-treatment and pharmacist). None of the respondents reported visiting sangomas or herbalists. Respondents with CND were significantly more likely to utilise public healthcare services, particularly public clinics, and less likely to utilise private healthcare services (p<0.05), reflecting repeat visits for the collection of medication. Other than this, the type of illness reported appeared to have no further effect on choice of healthcare provider.

A series of questions focused on the experiences of respondents who had accessed healthcare services in the last 6 months. Users of public clinics were more likely to report waiting times of over half an hour (p<0.01), consultation times of less than 10 minutes (p<0.01), more likely to access the services by walking (p<0.001), and less likely to incur expenses (p<0.001). Only about a third (32%) of those accessing private hospitals or clinics incurred expenses. The possession of medical aid was significantly likely (p<0.001) to influence the choice of a private healthcare provider, particularly private doctors and clinics.

The satisfaction ratings with receiving attention at the healthcare service provider, the helpfulness of the healthcare service provider, the waiting time, the willingness of staff to listen to concerns, and the healthcare service provider's understanding of needs and concerns were all significantly influenced by the type of healthcare service accessed (p<0.001) (Table 3). Satisfaction ratings were consistently lower for public hospitals, and particularly public clinics. Satisfaction ratings were generally lower for waiting times across all types of healthcare service providers.

**Table 3:** Comparison of satisfaction ratings by type of healthcare service provider (spell out).

	Private doctor or clinic	Public hospital	Public clinic	
Satisfaction with getting someone to attend to me (N = 243)	94.7%	86.1%	66.7%	p<0.001
Satisfaction with the helpfulness of the staff (N = 242)	94.7%	88.4%	63.5%	p<0.001
Satisfaction with the waiting time (N = 242)	86.3%	73.8%	45.7%	p<0.001
Satisfaction with willingness of staff to listen to concerns (N = 241)	92.6%	86.1%	72.8%	p<0.001
Satisfaction with staff's understanding of needs and concerns (N = 242)	92.6%	83.7%	71.4%	p<0.001

We asked all respondents (n = 1 102) what were the main problems they experienced in accessing healthcare from government clinics and hospitals. The top five problems (mentioned by over 75% of respondents) were long waiting times (24.5%), unfriendly staff or poor service (17.8%), lack of medication (17.3%), overcrowding (13.9%), and short consultation times (5%). Around 12% of respondents felt that there were no problems in accessing public healthcare services.

### Health-Seeking Behaviour

We asked the respondents a series of questions focused on three broad areas of health-seeking behaviour, namely reliance on family or friends for initial diagnosis and referral, utilisation of patient strategies when consulting doctors, and beliefs regarding the efficacy of traditional healers.

Reliance on family and friends for initial diagnosis and referral was high (76.7% and 81.8% respectively) although only around 10% (10.4%) reported exclusive reliance on the advice of family and friends for accessing healthcare services (Table 4). Respondents reported a low use of patient strategies; including partial disclosure of symptoms (16.3%) and rehearsal (15.9%), although almost a quarter (24.9%) indicated partial compliance with medical regimens.

**Table 4:** Health-seeking behaviour.

	N	%	Total
**Family and friends help interpret symptoms**	841	76.7%	**1 096**
**Family and friends advise where to seek healthcare**	899	81.8%	**1 099**
**Only seek healthcare when family and friends say so**	114	10.4%	**1 097**
**Do not always tell doctor all symptoms**	179	16.3%	**1 098**
**Rehearse before consulting doctors**	175	15.9%	**1 098**
**Do not always do what doctor says**	273	24.9%	**1 098**
**Doctors are more effective than traditional healers**	883	80.6%	**1 096**
**Traditional healers are respected in community**	490	44.8%	**1 095**

Belief in the efficacy of traditional healers was low, with the large majority of respondents (80.6%) feeling that formal medical institutions were more effective than traditional healers in treating illness. Less than half the respondents (44.8%) felt that traditional healers were well respected within their communities.

We asked the research participants a separate question about whether there were some diseases which could only be treated by traditional healers, and not by doctors. Slightly less than a third (31.8%) of respondents felt that there were some diseases which could only be treated by traditional healers. We grouped these responses into different categories, the top three being ‘witchcraft/curses/poisoning/evil spirits’ (23%), ‘fits/headache/mental illness’ (17.7%) and ‘HIV/AIDS’ (12.8%). Around 10% (9.9%) felt that various forms of CND (mostly ‘stroke’, or ‘high blood’ and to a lesser extent diabetes) could be treated properly only by traditional healers.

## Discussion

The prevalence of CND in this study population is double (32.5%) that reported in the South African Demographic and Health Survey (1998), which reported a 15.9% prevalence rate for hypertension in females [10]. The most noticeable feature of the findings is that over half the respondents (50.7%) in the current study reported having at least one CND, but only a third (33.3%) of these respondents accessed healthcare services in the last 6 months. The fact that 69% of respondents were using chronic treatment may suggest that respondents who actually accessed healthcare via the pharmacy to collect medication did not view this as healthcare access. However, this is unlikely as the pharmacy is located within the clinic, and thus a visit to the pharmacy would be seen as a visit to the clinic for those respondents who used a public clinic for this purpose. Rather, this finding suggests that most respondents with CND did not regularly comply with treatment regimens. This conclusion is supported by the fact that the overall healthcare utilisation rate for this study (24.3%) was the same as compared to the utilisation rate reported in the South African Demographic and Health survey [10].

It is unlikely that the low healthcare utilisation rate reported in the current study is due to poor availability or affordability of healthcare services in Soweto, as we found these to be high (86% and 82% respectively). The fact that around 16% of respondents felt that government clinics, which are free of charge in South Africa, are unaffordable suggests that indirect costs such as transportation and time-off work may prevent regular access to the clinics; a finding which has been reported elsewhere in South Africa [11]. This alone, however, cannot account for the low utilisation found in our study.

We can neither attribute the utilisation rate to dissatisfaction with healthcare services, since our satisfaction ratings, although significantly lower for public than private healthcare facilities, were still above 80% (with the exception of waiting times). The fact that almost a quarter of respondents (24.9%) reported partial compliance with medical regimens suggests that a large portion of respondents strategically select their intake of medication and their subsequent visits to the clinic to collect medication.

The coincidence of the self reported non-utilisation of traditional healers with high proportions of responses indicating knowledge of the location and cost of traditional healers within the neighbourhood (32.1%) warrants further consideration. Furthermore, a sizeable proportion of respondents (19.4%) disagreed with the statement that doctors were more effective than traditional healers in treating illness, and, in a separate question, almost a third of respondents (31.8%) felt that there were some diseases which could only be treated by traditional healers. In this instance it is difficult to attribute the low reported utilisation of traditional healers to social desirability bias, given its apparent absence in the other questions. The low utilisation of traditional healers is similar to other findings in South Africa [12], [13], [14].

These findings suggest a demarcation between the utilisation of traditional healers for treating conditions identified as chronic disease, and the use of traditional healers for other purposes. The findings from our study indicate that only 10% of those respondents who stated that traditional healers were more effective than doctors specified that traditional healers could more effectively treat CNDs. The disparity in figures relating to utilisation of traditional healers for the treatment of conditions conceived as disease on one hand and general knowledge on the other, may indicate that traditional healers are often consulted for non-biomedical conditions, such as protection from witchcraft and poisoning. Given the specific Sowetan cultural background, fits and headaches may also be interpreted as primarily spiritual or social in nature [15].

## Conclusion

Our study highlights the potentially poor management of CND due to infrequent access of health care despite it being accessible and affordable. The low utilisation of healthcare services indicates that the full realisation of PHC in South Africa has not been attained. In particular, our findings illustrate that a large proportion of those with CND do not access regular treatment most probably out of choice, as availability, affordability, and even acceptability (via high satisfaction ratings) of formal health services were generally high. More research as to the barriers to regular health care attendance and adherence to chronic medication is urgently warranted.

For African countries undergoing transition, the South African scenario highlights the need to develop innovative strategies for the implementation of PHC. African countries which have previously demonstrated the political will to develop and implement PHC have often been hampered by weak economies and limited budgets [16]. In this context, community based organisations, particularly faith based organisations (FBOs) have played an important role in providing health care services, often drawing from long histories of intervention [17]. Often competing with FBOs, but also functioning as *de facto* health care provider in many African countries are traditional healers, who in many African populations embody generally accepted world views [18].

For South Africa, community participation in community health centres is a key principle of the PHC approach, but developing community commitment to PHC remains a challenge [19]. It appears from our findings that both demand-side and supply-side features of PHC in South Africa must be addressed to ensure full and satisfying utilisation of formal healthcare facilities. In this regard, the recognition of the potential role played by community partnerships in the implementation of PHC has been a key factor informing new approaches to revitalise PHC in South Africa and Gauteng. In particular, regions of Gauteng have recently been identified as sites for the implementation of Community Oriented Primary Care (COPC), entailing the development of service units within communities largely resourced by Community Health Workers (CHWs) and community based organisations [20]. Primary healthcare workers and general practitioners have an important role to play within COPC not only in ensuring the congruence of formal healthcare services with community needs, but also in designing and implementing community-based interventions based on in depth assessments of community healthcare needs [21]. Such an approach heralds the return of the optimism marking the birth of PHC on South African soil [3]. In the ongoing management of CND, it should be remembered that community and group self-management programmes have proved effective in maintaining social connectedness and community support in the management of CND, especially in the context of low formal healthcare utilisation [22].

The successful implementation of the COPC approach marks the beginning of healthcare access studies which do not focus primarily on the utilisation of clinical services, but rather on the participation of individuals with CND in community based healthcare structures. Such studies should further investigate how healthcare beliefs are formed and how they interact with formalised knowledge of CND in the management practices of women with CND. Ensuring the relevance of the PHC approach for South African communities will require innovative research and leadership that is responsive to community social dynamics with regard to the perception and experience of CND.
